# MCMICRO: a scalable, modular image-processing pipeline for multiplexed tissue imaging

**DOI:** 10.1038/s41592-021-01308-y

**Published:** 2021-11-25

**Authors:** Denis Schapiro, Artem Sokolov, Clarence Yapp, Yu-An Chen, Jeremy L. Muhlich, Joshua Hess, Allison L. Creason, Ajit J. Nirmal, Gregory J. Baker, Maulik K. Nariya, Jia-Ren Lin, Zoltan Maliga, Connor A. Jacobson, Matthew W. Hodgman, Juha Ruokonen, Samouil L. Farhi, Domenic Abbondanza, Eliot T. McKinley, Daniel Persson, Courtney Betts, Shamilene Sivagnanam, Aviv Regev, Jeremy Goecks, Robert J. Coffey, Lisa M. Coussens, Sandro Santagata, Peter K. Sorger

**Affiliations:** 1 Ludwig Center for Cancer Research at Harvard, Harvard Medical School, Boston, MA USA; 2 Laboratory of Systems Pharmacology, Harvard Medical School, Boston, MA USA; 3 Klarman Cell Observatory, Broad Institute of MIT and Harvard, Cambridge, MA USA; 4 Department of Biomedical Informatics, Harvard Medical School, Boston, MA USA; 5 Image and Data Analysis Core, Harvard Medical School, Boston, MA USA; 6 Vaccine and Immunotherapy Center, Massachusetts General Hospital, Harvard Medical School, Boston, MA USA; 7 Biomedical Engineering, Oregon Health and Science University, Portland, OR USA; 8 Department of Medical Oncology, Dana-Farber Cancer Institute, Boston, MA USA; 9 Department of Biology, Brigham Young University, Provo, UT USA; 10 Epithelial Biology Center, Vanderbilt University Medical Center, Nashville, TN USA; 11 Department of Cell and Developmental Biology, Vanderbilt University School of Medicine, Nashville, TN USA; 12 Knight Cancer Institute, Oregon Health and Science University, Portland, OR USA; 13 Department of Cell, Developmental and Cancer Biology, Oregon Health and Science University, Portland, OR USA; 14 Department of Biology, Howard Hughes Medical Institute, Massachusetts Institute of Technology, Cambridge, MA USA; 15 Division of Gastroenterology, Hepatology, and Nutrition, Department of Medicine, Vanderbilt University Medical Center, Nashville, TN USA; 16 Department of Pathology, Brigham and Women’s Hospital, Harvard Medical School, Boston, MA USA; 17 Department of Systems Biology, Harvard Medical School, Boston, MA USA; 18 Present Address: Institute for Computational Biomedicine and Institute of Pathology, Faculty of Medicine, Heidelberg University Hospital and Heidelberg University, Heidelberg, Germany; 19 Present Address: Genentech, South San Francisco, CA USA

**Keywords:** Image processing, Software, Systems biology

## Abstract

Highly multiplexed tissue imaging makes detailed molecular analysis of single cells possible in a preserved spatial context. However, reproducible analysis of large multichannel images poses a substantial computational challenge. Here, we describe a modular and open-source computational pipeline, MCMICRO, for performing the sequential steps needed to transform whole-slide images into single-cell data. We demonstrate the use of MCMICRO on tissue and tumor images acquired using multiple imaging platforms, thereby providing a solid foundation for the continued development of tissue imaging software.

## Abstract (web summary)

MCMICRO is a modular and open-source computational pipeline for transforming highly multiplexed whole-slide images of tissues into single-cell data. MCMICRO is versatile and can be used with CODEX, mxIF, CyCIF, mIHC and H&E staining data.

## Main

Highly multiplexed imaging of tissues and tumors makes it possible to measure the levels and localization of 20–100 proteins at subcellular resolution in a preserved spatial environment (see Supplementary Table 1 for references). These proteins are usually detected using antibodies, often in conjunction with stains such as Hoechst 33342 or hematoxylin and eosin (H&E). Cell identities, phenotypes and states can then be identified on the basis of staining intensities and patterns. This makes image-based single-cell analysis a natural complement to spatial and single-cell transcriptomics^1–3^ and promises to augment traditional histopathological diagnosis of disease^4–6^.

Computational analysis of highly multiplexed tissue images presents challenges not readily addressed with existing software. This is particularly true of whole-slide tissue imaging (WSI), in which specimens as large as 5 cm^2^ yield up to 1 terabyte of data (a 50-plex 4 cm^2^ at 0.3 µm lateral resolution), 10^6^ to 10^7^ cells and resolvable structure from 100 nm to over 1 cm. The US Food and Drug Administration mandates WSI for diagnostic histopathology^7^, and it is essential for accurately quantifying mesoscale tissue structures^8^.

Four primary challenges must be overcome to make computational analysis of high-plex WSI routine and reproducible: (1) data acquired in multiple image fields must be assembled precisely into large mosaic images encompassing the whole specimen and multiple imaging cycles; (2) full-resolution images must be made available in conjunction with numerical results; (3) images must be subdivided (segmented) into single cells—a difficult task when cells are densely crowded and nuclei have irregular morphologies; (4) diverse image-processing algorithms and data types must be harmonized across research groups and programming languages. Analogous challenges in genomics have been addressed using computational pipelines such as Seurat, Scanpy and Cumulus (see Supplementary Table 2 for references), platforms such as Galaxy^9^ that make use of software containers (for example, Docker^10^) and formal domain-specific workflow languages such as Nextflow^11^. These tools simplify the task of creating, maintaining and improving computational pipelines, including in the cloud.

In this paper, we describe MCMICRO (Multiple Choice MICROscopy), a scalable, modular and open-source image-processing pipeline that leverages Docker/Singularity containers^10,12^ and is implemented in Nextflow^11^ and Galaxy^9^. The Nextflow implementation uses a plain-text configuration file to simplify addition, management and execution of modules and a command line interface; Galaxy uses Conda environments for package management and a graphical user interface (GUI). A diverse community of laboratories including those involved in the Human Tumor Atlas Network (HTAN; https://humantumoratlas.org)^13^ maintains and develops MCMICRO. Documentation, source code and video tutorials are available at mcmicro.org, and help is available via the image.sc forum.

**Fig. 1:**
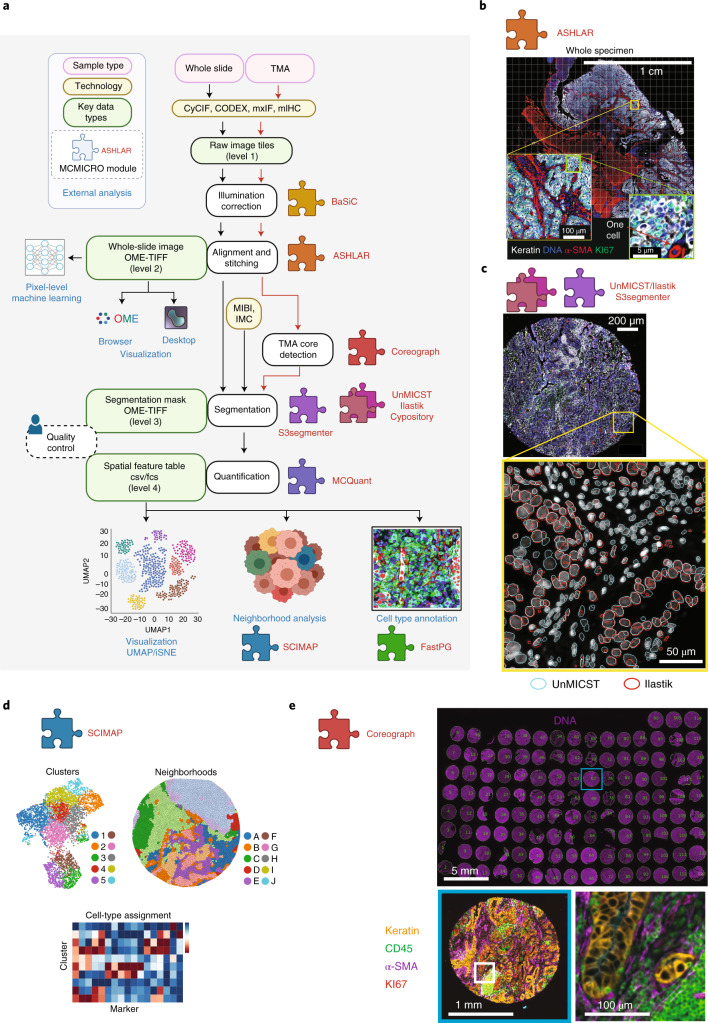
Overview of the MCMICRO pipeline and key data types. Modules highlighted in bold red were developed and/or containerized as part of this study. **a**, A schematic representation of a canonical workflow for end-to-end image processing of multiplexed whole-slide and TMA data using MCMICRO. Shown is a flow of inputs (pink rectangles) from imaging instruments (yellow rectangles) through image-processing steps (white rectangles) that are implemented in software modules (puzzle pieces) to produce key data types (green rectangles). Data flows associated with a whole-slide image and TMA are represented with black and red arrows, respectively. **b**–**e**, Highlights of individual software modules incorporated into MCMICRO. **b**, ASHLAR is used to stitch and register individual CyCIF image tiles with subcellular accuracy (yellow zoom-in). This panel depicts a whole-slide, 484 tile (22 × 22) mosaic t-CyCIF image of a human colorectal cancer in four channels: Hoechst 33342-stained nuclear DNA (blue) and antibody staining against α-smooth muscle actin (α-SMA; red), the Ki-67 proliferation marker (green) and cytokeratin (white). **c**, Two different segmentation masks computed by UnMICST (blue) and Ilastik (red) overlaid on an image of nuclei from an EMIT TMA core (single experiment). **d**, SCIMAP enables single-cell clustering, neighborhood analysis and cell-type assignment on the basis of patterns of marker expression. **e**, A CyCIF image of an EMIT TMA dearrayed using Coreograph to identify individual cores, which are subsequently extracted and analyzed in high resolution. Below, a five-color image of a single lung adenocarcinoma core is shown for channels corresponding to Hoechst 33342-stained DNA (white), cytokeratin (orange), the immune-cell marker CD45 (green), α-SMA (magenta) and Ki-67 (red).

To create MCMICRO, we wrote five new software packages, three of which are described here (Coreograph for subdividing tissue microarrays (TMAs); Cypository for segmenting cytoplasm and SCIMAP for spatial data analysis). Two complex packages are described elsewhere (universal models for identifying cells and segmenting tissue (UnMICST^14^) and alignment by simultaneous harmonization of layer/adjacency registration (ASHLAR^15^)). We also reimplemented several MATLAB algorithms (MCQuant^16^ for quantifying marker intensities and computing morphology metrics and S3segmenter^17^ for watershed segmentation and spot detection); we also integrated existing algorithms written by others (for example, BaSiC^18^, Ilastik^19^ and FastPG (a variant of PhenoGraph) (see Methods for references)). All algorithms were tuned to manage very large files and containerized to abstract away language-specific dependencies (Methods). MCMICRO can run multiple algorithms in parallel, allowing their performance to be compared directly, which is particularly helpful for tasks such as segmentation. In common with other bioinformatics pipelines, MCMICRO complements rather than replaces desktop and server-deployed tools, particularly for visualization. Model training and parameter adjustment for tasks such as segmentation can take place locally, with visualization using Napari, QuPath, OMERO and histoCAT (Fig. 1a and Supplementary Table 2), followed by running the model on large datasets in the cloud.

To accelerate algorithm benchmarking and training, we generated a set of freely available Exemplar Microscopy Images of Tissues and Tumors (EMIT) comprising multiplexed images of a TMA with 120 1.5 mm cores from 34 types of cancer, non-neoplastic disease and normal tissue (Supplementary Fig. 1). EMIT images were processed using MCMICRO, with all intermediate steps documented on Sage Bionetworks Synapse as described in the dataset section of mcmicro.org. Clustering of cancers by type demonstrates effective segmentation across a wide range of specimens and generation of meaningful single-cell data (Supplementary Fig. 2).

Processing multiplexed WSI data starts with acquisition of image tiles (fields) in a BioFormats-compatible format (Level 1 data; Fig. 1a)^20^. Each tile is typically a megapixel multichannel image; ~10^3^ tiles are needed to cover a large specimen at subcellular resolution. Tiles are corrected for uneven illumination using BaSiC, then stitched and registered across channels using ASHLAR to generate a fully assembled, multichannel mosaic image in OME-TIFF (open microscopy environment-tagged image file format) format (Level 2 or 3 data depending on the extent of quality control; Fig. 1b). Mosaic length scales vary 10^5^-fold from the smallest resolvable feature to the largest, and can be visualized interactively using web-based tools such as OMERO or desktop software (Supplementary Table 2). A segmentation mask (Level 3 data) is used to subdivide images into cells, available for human inspection (Fig. 1c). Segmentation is also facilitated by a ‘classifier zoo’ trained on EMIT TMA data.

Following segmentation, the staining intensity in each channel, cell morphology (size, eccentricity and so on) and other characteristics are computed on a per-cell basis to generate a Spatial Feature Table (Level 4 data), which is analogous to a count table in scRNAseq. Spatial Feature Tables can be visualized using dimensionality reduction tools such as tSNE or UMAP, processed to identify cell types and used for neighborhood analysis (for example, with SCIMAP; Fig. 1d). It is also possible to skip segmentation and perform analysis directly on images using pixel-level deep learning. Regardless of how data flows through MCMICRO, provenance is maintained by recording the identities, version numbers and parameter settings for each module, ensuring reproducibility (Supplementary Fig. 3).

Some types of imaging data require additional processing. TMAs, for example, must be subdivided into constituent 0.3 to 2 mm diameter ‘cores’. Coreograph accomplishes this using a U-Net deep learning architecture^21^ (Fig. 1e). Each core is its own multichannel image that can be further processed by MCMICRO (Supplementary Fig. 4). The robustness of neural networks makes it possible for Coreograph to identify cores even in highly distorted TMAs.

**Fig. 2:**
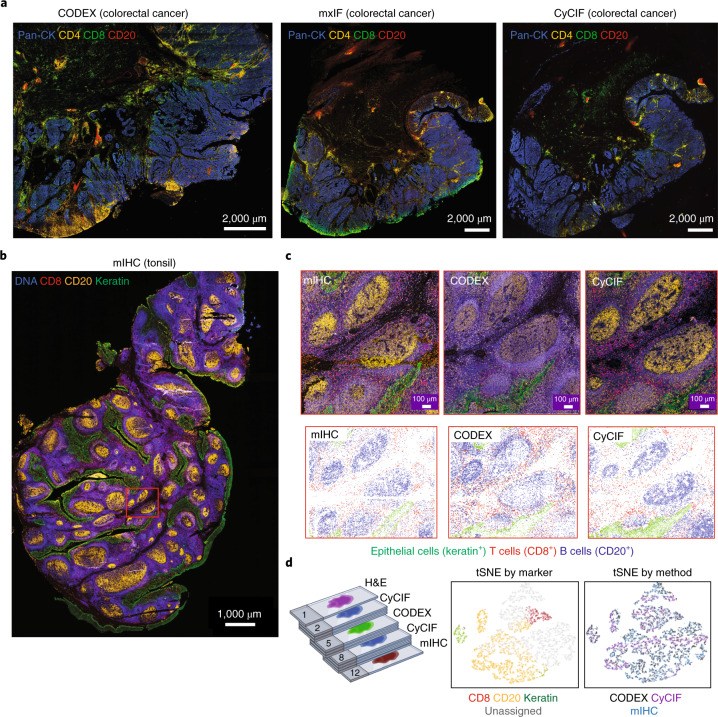
Exemplary whole-slide images processed using MCMICRO. **a**, Selected channels are shown from three exemplary high-plex images from colorectal cancer using CODEX, MxIF and CyCIF; pan-cytokeratin is depicted in blue; CD4 in yellow; CD8 in green and CD20 in red. **b**, Selected channels are shown from a high-plex image of human tonsil using mIHC with Hoechst 33342 stain in blue (representing DNA), CD20 in orange, keratin in green and CD8 in red. **c**, Upper panels: selected fields of view from mIHC, CODEX and CyCIF images of the tonsil specimen shown in **b** (the selected field is highlighted by the red rectangle). Lower panels: centroids for the single-cell segmentation mask for the three fields of view shown above and colored by marker expression to identify cell types. Epithelial cells of the tonsil mucosa stain positive for pan-cytokeratin (green), cytotoxic T cells stain positive for CD8 (red) and B cells stain positive for CD20 (blue). **d**, Schematic of the comparative analysis of a single tonsil specimen and two tSNE plots for CODEX, CyCIF and mIHC data (shown in **c**) demonstrating clustering by marker expression (left) but not imaging technology (right).

To demonstrate the technology-agnostic capabilities of MCMICRO implementations in Galaxy and Nextflow (Supplementary Fig. 5), we analyzed WSI data from an FFPE colorectal cancer resection (Fig. 2a) and human tonsil (Fig. 2b) using images collected at four different institutions with five technologies: codetection by indexing (CODEX), multiplex immunofluorescence (mxIF), cyclic immunofluorescence (CyCIF), multiplexed immunohistochemistry (mIHC) and H&E staining; we also processed publicly available imaging mass cytometry (IMC) and multiplexed ion beam imaging (MIBI) data (Supplementary Table 1). Data processing was performed using cloud compute nodes provided either by Amazon Web Services (AWS), the Google Cloud Platform or a Linux-based institutional cluster running the SLURM workload manager. MCMICRO provides detailed information on time, memory and central processing unit (CPU) use, making it straightforward to provision necessary computational resources (Supplementary Fig. 4).

Image tiles from a variety of microscopes and acquisition technologies were subjected to stitching, registration and illumination correction using ASHLAR and BaSiC to generate level 2 image mosaics that were inspected on a local workstation using Napari and in the cloud using OMERO **(**Fig. 2a–c**)**. Images were segmented and staining intensities computed on a per-cell basis using MCQuant. Cell types were visualized in a tissue context for epithelial cells of the tonsil mucosa (keratin^+^ panCK^+^), cytotoxic T cells (CD8^+^) and B cells (CD20^+^) (Fig. 2c). Visual inspection of stitched and registered CyCIF, CODEX and mIHC images and derived data revealed accurate stitching and registration, as well as creation of reasonable segmentation masks and correctly formatted Spatial Feature Tables. When visualized by tSNE, data were separated by marker expression, not by imaging technology (Fig. 2d), demonstrating consistency in data processing.

A few algorithms in MCMICRO (for example, ASHLAR and BaSiC) are tissue- and technology-agnostic and require little, if any, tuning or modification. The performance of other algorithms (for example, UnMICST, Cypository and Ilastik) is dependent on the properties of their learned models, which can be tissue-specific. Looking forward, we expect rapid development of modules focused on four primary tasks: (1) image segmentation, (2) image quality control, (3) assignment of cell types and states on the basis of marker intensities and cell morphologies and (4) methods for quantifying spatial relationships and neighborhoods. A roadmap covering some of these developments is available at mcmicro.org. However, we do not anticipate that MCMCRO users will need to manage an endless proliferation of algorithms; multiple research consortia are sponsoring evaluation efforts to identify best practices. MCMICRO will provide a technical foundation for such evaluations.

Adoption of MCMICRO will enable community-wide development of FAIR (findable, accessible, interoperable and reusable)^22^ workflows for analysis of the large tissue images being generated by international consortia and individual laboratories. MCMICRO works with six of the primary image acquisition technologies in use today, and is designed to accommodate future BioFormats/OME-compatible approaches. The pipeline is based on widely accepted software standards and interoperates with any programming language through the use of software containers, making it possible for developers to add new modules and models. Our experience also suggests that new users can master either the NextFlow command line interface or the Galaxy GUI with a day of training.

## Methods

### Tissue samples

A deidentified tonsil specimen from a 4-year-old female of European ancestry was procured from the Cooperative Human Tissue Network (CHTN), Western Division, as part of the HTAN SARDANA Trans-Network Project. Regulatory documents including Institutional Review Board (IRB) protocols, data use agreements and tissue use agreements were in place to ensure regulatory compliance. Standard protocols for tissue procurement and fixation were followed; a detailed protocol can be found at the link provided in Table 1. Sections were cut from a common formalin-fixed paraffin embedded (FFPE) block at a thickness of 5 µm and mounted onto Superfrost Plus glass microscope slides (Fisher Scientific, catalog no. 12-550-15) for CyCIF and mIHC or mounted on poly-l-lysine-coated coverslips (Electron Microscopy Sciences, catalog no. 72204-01; slides and FFPE sections were prepared following instructions in the Akoya Biosciences CODEX User Manual Rev B.0, Chapter 3. Coverslip Preparation and Tissue Processing) for CODEX. A set of FFPE tissue sections was received by participating HTAN Centers (CHTN, Harvard Medical School (HMS), Broad Institute, and Oregon Health and Science University (OHSU)) as indicated in Table 2, allowing Centers to generate a comparable spatial cell census using each Center’s imaging method of choice. CHTN performed H&E staining on the first section, which was subsequently imaged at HMS.

**Table 1:** Experimental protocols

Category	Center	Protocols.io link
Protocol (biospecimen)	CHTN	Tissue procurement and fixation in 10% neutral buffered formalin 10.17504/protocols.io.6y4hfyw
Protocol (characterization)	HMS	H&E 10.17504/protocols.io.bsi8nchw
Protocol (characterization)	HMS	FFPE tissue pretreatment before tissue-CyCIF on Leica Bond RX v.2 10.17504/protocols.io.bji2kkge
Protocol (characterization)	HMS	Tissue-CyCIF 10.17504/protocols.io.bjiukkew
Protocol (characterization)	Broad Institute	CODEX 10.17504/protocols.io.brznm75e
Protocol (characterization)	OHSU	mIHC https://www.protocols.io/view/mihc-staining-ohsu-coussens-lab-sop-tnp-sardana-bcdpis5n

As a part of the HTAN effort, all protocols and methods are deposited with protocols.io.

**Table 2:** Sample information for tonsil image data in Fig. 2

Section number	Section thickness (µm)	Center	Assay
WD-75684-01	5	CHTN	H&E
WD-75684-02	5	HMS	CyCIF
WD-75684-05	5	Broad Institute	CODEX
WD-75684-08	5	HMS	CyCIF
WD-75684-12	5	OHSU	mIHC

For the EMIT dataset, human tissue specimens (from 42 patients) were used to construct a multitissue microarray (HTMA427) under an excess (discarded) tissue protocol approved by the IRB at Brigham and Women’s Hospital (BWH IRB 2018P001627). Two 1.5-mm-diameter cores were acquired from each of 60 tissue regions with the goal of acquiring one or two examples of as many tumors as possible (with matched normal tissue from the same resection when that was feasible), as well as several non-neoplastic medical diseases involving acute inflammation (for example diverticulitis and appendicitis), and secondary lymphoid tissues such as tonsil, spleen and lymph nodes. Overall, the TMA contained 120 cores plus 3 additional ‘marker cores’, which are cores added to the TMA in a manner that makes it possible to orient the TMA in images.

### CyCIF staining and imaging

The CyCIF method involves iterative cycles of antibody incubation, imaging and fluorophore inactivation as described previously^8^. A detailed protocol can be found on protocols.io as shown in Table 1, with detailed antibody information available in Supplementary Tables 3 and 4. CyCIF images are 36-plex whole-slide images collected using a ×20 magnification, 0.75 numerical aperture objective with 2 × 2 pixel binning, yielding images of pixel size at 0.65 µm per pixel. The image comprises 416 and 350 image tiles for WD-75684-02 and WD-75684-08, respectively, each with four channels, one of which is always Hoechst to stain DNA in the nucleus.

### CODEX staining and imaging

Coverslips were prepared following the FFPE tissue staining protocols in the Akoya Biosciences CODEX User Manual (Sections 5.4–5.6). Briefly, 5 μm FFPE tissue sections were cut onto poly-l-lysine-coated coverslips and baked for 20–25 min at 55 °C. Sections were cooled briefly before deparaffinization and washed for 5 min each as follows: twice in xylene; twice in 100% ethanol; once in 90%, 70%, 50% and 30% ethanol and twice in deionized water. Sections were moved to 1× Citrate Buffer (Vector Laboratories, catalog no. H-3300) and antigen retrieval was performed in a Tinto Retriever Pressure Cooker (BioSB, BSB 7008) at high pressure for 20 min. Sections were washed briefly in deionized water before being left to incubate in deionized water at room temperature for 10 min. Sections were washed briefly twice in Hydration Buffer (Akoya), then were left to incubate in Staining Buffer (Akoya) at room temperature for 20–30 min. Antibody cocktail (200 μl per section) was prepared according to the manufacturer’s instructions. Sections were covered with the 200 μl Antibody Cocktail and left to incubate at room temperature for 3 h in a humidity chamber. Sections were washed twice in Staining Buffer for 2 min, and then fixed with a mixture of 1.6% paraformaldehyde in Storage Buffer (Akoya) for 10 min. Sections were washed briefly three times in 1× PBS, and then washed in ice-cold methanol for 5 min before being washed again three times in 1× PBS. Sections were stained with 190 μl of a mixture of 20 μl Fixative Reagent (Akoya) and 1 ml 1× PBS, after which they were left to incubate at room temperature for 20 min. Sections were washed briefly three times in 1× PBS and stored in Storage Buffer at 4 °C until the assay was ready to be run.

### Running the CODEX assay

A 96-well plate of reporter stains with Nuclear Stain (Akoya) was prepared according to Akoya Biosciences CODEX User Manual (Sections 7.1–7.2). Stained tissue sections were loaded onto the CODEX Stage Insert (Akoya) and the Reporter Plate was loaded into the CODEX Machine. The onscreen prompts were followed, and the section was stained manually with a 1:2,000 Nuclear Stain in 1× CODEX Buffer (Akoya) for 5 min before proceeding by following the onscreen prompts. Imaging was performed on a Zeiss Axio Observer with a Colibri 7 light source. Emission filters were BP 450/40, BP 550/100, BP 525/50, BP 630/75, BP 647/70, BP 690/50 and TBP 425/29 + 514/31 + 632/100, and dichroic mirrors were QBS 405 + 492 + 575 + 653, TFT 450 + 520 + 605, TFT 395 + 495 + 610 and TBS 405 + 493 + 575, all from Zeiss. Overview scans were performed at ×10 magnification, after which 5 × 5 field of view regions were acquired using a Plan-Apochromat ×20/0.8 M27 Air objective (Zeiss, catalog no. 420650-9902-000). Magnification images (×20) were acquired with a 212 × 212 nm pixel size using software autofocus repeated every tile before acquiring a 17 plane z-stack with 0.49 µm spacing. Tiles were stitched using a 10% overlap.

### mIHC staining and imaging

The mIHC platform described herein involves wet and dry laboratory techniques that have been previously described^23,24^. A detailed protocol utilized for the current study is available on protocols.io (Table 2). mIHC involves a cyclic staining process optimized for FFPE tissues with panels of antibodies (12–29 per panel) designed to interrogate both lymphoid and myeloid compartments of the immune system. Each antibody is stained singularly and culminates with whole-slide digital imaging, as the staining chemistry utilizes a single chromogen and brightfield imaging. Hematoxylin staining at the beginning and end of the antibody panel is used for nuclear identification in the computational pipeline. Whole-slide images are scanned at ×20 magnification, with 0.5 μm per pixel.

### Pipeline implementation

MCMICRO was implemented in Nextflow, which was chosen for its natural integration with container technologies such as Docker and Singularity, its automatic provenance tracking and parallelization of image-processing tasks and its ability to specify module dependencies that may change at runtime^11^. To make the MCMICRO pipeline more widely available, we have also integrated it with the Galaxy computational workbench, which is used daily by thousands of scientists across the world for a wide array of biomedical data analyses (Supplementary Fig. 5)^9^.

### Illumination correction

BaSiC is a Fiji/ImageJ plugin for background and shading correction, producing high accuracy while requiring only a few input images^18^. We containerized the tool, allowing it to be executed without an explicit installation of ImageJ.

### Image stitching and registration using ASHLAR

ASHLAR (Alignment by Simultaneous Harmonization of Layer/Adjacency Registration) is a Python package for efficient mosaicing and registration of highly multiplexed imagery^15^. It performs stitching and registration on cyclic immunofluorescence images using data from nuclear stains (typically Hoechst 33342). The overall strategy is to: (1) align tile images from the first cycle edge-to-edge with their nearest neighbors (mosaicing) using phase correlation on the nuclear marker channel; (2) for the second and subsequent cycles, align each tile to the greatest overlapping tile from the first cycle (registration), using phase correlation on the nuclear marker channel and retain the corrected stage coordinates, rather than the actual merged images; (3) use the corrected coordinates to assemble a single image covering the entire imaged area, including all channels from all cycles. This approach minimizes the compounding of alignment errors across tiles and cycles as well as temporary storage requirements for intermediate results.

### Coreograph

Coreograph was newly developed for MCMICRO and has not been published elsewhere. It is implemented for the first time in MCMICRO. Its function is to split, or ‘dearray,’ a stitched TMA image into separate image stacks per core. It employs a semantic segmentation preprocessing step to assist with identifying cores that are dimmed or fragmented, which is a common issue. We trained a deep, fully connected, network on two classes—core tissue and background—using the popular UNet^21^ architecture for semantic segmentation. Training data consisted of cores that were well separated, as well as cores that were merged and/or fragmented, which allowed for handling situations where sample integrity was highly heterogeneous. Once cores had been accentuated in the form of probability maps, they were cropped from the stitched image on the basis of their median diameter and saved as a TIFF stack. In situations where the cores were too clumped, the median diameter was used to set the size of a Laplacian of Gaussian (LoG) kernel to identify local maxima from the probability maps.

### UnMICST

UnMICST^14^ is a module in MCMICRO that aids in improving downstream segmentation accuracy by generating per class probability maps to classify each pixel with a certain amount of confidence. Analogously to Coreograph, it employs a UNet architecture (above). Previously, a similar UNet model was trained for nuclei segmentation to recognize two classes in Hoechst 33342-stained tonsil tissue (nucleus contours and background). Here, we train a three-class model to extract nuclear centers, nucleus contours and background from manually annotated lung, tonsil, prostate and other tissues to ascribe a variety of nucleus shapes. Realistic augmentations, in addition to conventional on-the-fly transformations, were included by deliberately defocusing the image and increasing the exposure time of the camera to simulate focus and contrast augmentations, respectively. Training was performed using a batch size of 24 with the Adam Optimizer and a learning rate of 0.00003 until the accuracy converged. Segmentation accuracy was estimated by counting the fraction of cells in a held out test set that passed a sweeping intersection of union metric.

### Ilastik tissue segmentation

Like UnMICST, Ilastik^19^ assigns each pixel a probability of belonging to predetermined classes (for example, cell nucleus, membrane and background). MCMICRO relies on Ilastik’s pixel classification module for training and subsequent batch processing using a random forest classifier. Ilastik classifier training in MCMICRO is completed in several steps. First, regions of interest (ROIs) with a user-defined width and height are cropped randomly from the WSI. Second, the ROIs are annotated manually by the user on a local machine via Ilastik’s GUI. Third, to ensure tissue portions are accurately represented in cropped images, Otsu’s method is used to identify a global threshold across the WSI for a particular channel of interest (for example, nuclear staining). Finally, the user exports the cropped sections that contain the desired proportion of pixels above the previously determined threshold. Upon completion of the random forest training, whole-slide classifier predictions are deployed in headless mode (no GUI) for batch processing of large datasets within MCMICRO.

### Cypository

Cypository is an instance segmentation module, implemented in PyTorch and on the basis of Mask R-CNN architecture. The underlying two-class model was pretrained using the Common Object in Context dataset and then refined to distinguish whole cells from background on the basis of a cell membrane channel stained with wheat germ agglutinin. Training was performed using a batch size of four, a learning rate of 0.005, a momentum of 0.9 and weight decay of 0.0005 over five epochs. Unlike UnMICST, the output of Cypository is both a label mask and bounding boxes that encompasses each detected cell; the label mask is compatible with downstream modules in MCMICRO.

### Watershed segmentation via S3segmenter

S3segmenter was newly implemented for the MCMICRO pipeline and comprises a custom marker-controlled watershed algorithm to identify nuclei from the probability maps generated by UnMICST and Ilastik. Watershed markers are obtained by convolving a LoG kernel, followed by a local maxima search across the image to identify seed points. The size of the LoG kernel and local maxima compression are tunable parameters dependent on the expected nuclei diameters in the image. As a byproduct, this method identifies false positive segments in the image background. These false positives were excluded by comparing their intensities to an Otsu-derived threshold calculated either on the raw image or on the probability map. S3segmenter currently offers three alternative methods for cytoplasm segmentation. First, traditional nonoverlapping rings (annuli) with user-defined radius are used around each nucleus. Second, a Euclidean distance transform is computed around each nucleus and masked with a user-specified channel, reflecting the overall shape of the whole tissue sample. An autofluorescence channel can be chosen if the signal-to-image background ratio is sufficiently high. Third, the cytoplasm is segmented using a marker-controlled watershed on the grayscale-weighted distance transform, where the segmented nuclei are markers and the grayscale-weighted distance transform is approximated by adding scaled versions of the distance transform and raw image together. This method is conceptually similar to that found in the CellProfiler Identify Secondary Objects module^25^. S3segmenter is also capable of detecting puncta by convolving a small LoG kernel across the image and identifying local maxima. Once nuclei and cytoplasm segmentation are complete, labeled masks for each region are exported as 32-bit TIFF images. Two channel TIFF stacks consisting of the mask outlines and raw image are also saved so that segmentation accuracy can be easily visually assessed.

### MCQuant

Semantic segmentation in MCMICRO produces 32-bit masks, which are used to quantify pixel intensity (that is, protein expression) on multiplexed WSI for cytoplasm and nuclei. Quantification in MCMICRO is carried out using scikit-image—a popular Python-based image analysis library—and values of cellular spatial features are calculated for unique cells (cytoplasm and nuclei), in addition to their mean pixel intensity (protein expression). The resulting spatial feature tables are exported as comma-separated value (CSV) files for subsequent data analysis analogous to histoCAT^16^, which is implemented in MATLAB.

### SCIMAP

The spatial feature tables produced by MCMICRO can be used to perform a variety of single-cell, spatially resolved analyses. SCIMAP is a Python-based single-cell spatial analysis toolkit designed to work with large datasets. We incorporated SCIMAP into MCMICRO to perform unsupervised clustering (Leiden clustering^26^, Phenograph^27^, KMeans) for identification of cell types, and also spatial clustering to identify recurrent cellular neighborhoods^28^. The SCIMAP module outputs CSV files containing cluster annotations, as well as heatmaps and UMAP plots for cluster visualization. In addition, the module outputs an AnnData object that can be imported readily for further SCIMAP analysis in Jupyter notebooks and visualization with napari. The AnnData object is compatible with well-known single-cell toolkits such as Scanpy^29^ and Seurat^30^, allowing for seamless integration of imaging data with other single-cell modalities.

### FastPG

FastPG^31^ is a C++ implementation of the popular Phenograph method for clustering single-cell data. The implementation scales incredibly well for datasets with millions of cells—such as those routinely encountered in whole-slide imaging—often leading to an order of magnitude faster runtimes than the original Phenograph. Like SCIMAP, FastPG takes as input the spatial feature tables produced by MCMICRO and outputs an assignment of individual cells to clusters in the marker expression space.

### Reporting Summary

Further information on research design is available in the Nature Research Reporting Summary linked to this article.

## Online content

Any methods, additional references, Nature Research reporting summaries, source data, extended data, supplementary information, acknowledgements, peer review information; details of author contributions and competing interests; and statements of data and code availability are available at 10.1038/s41592-021-01308-y.

## Supplementary information

Supplementary Information Supplementary Figs. 1–5 and Tables 1–5.

Reporting Summary

## Data Availability

All EMIT and exemplar images are available at https://mcmicro.org/datasets.html.

## Code availability

All software and code that produced the findings of the study, including all main and supplemental figures, are available at https://github.com/labsyspharm/mcmicro.
