# Supplementary material for: MCMICRO: a scalable, modular image-processing pipeline for multiplexed tissue imaging
Source: Nat Methods. 2021 Nov 25;19(3):311–5. doi: 10.1038/s41592-021-01308-y (PMC8916956; doi:10.1038/s41592-021-01308-y)
Supplement: Supplementary file 1 — Supplementary Figs. 1–5 and Tables 1–5. [file 41592_2021_1308_MOESM1_ESM.pdf]

nature portfolio

https://doi.org/10.1038/s41592-021-01308-y

Supplementary information

MCMICRO: a scalable, modular image-processing pipeline for multiplexed tissue imaging

In the format provided by the authors and unedited

Figure S1

The EMIT dataset spanning 123 tissue cores across 34 cancer, non-neoplastic diseases, and normal tissue type

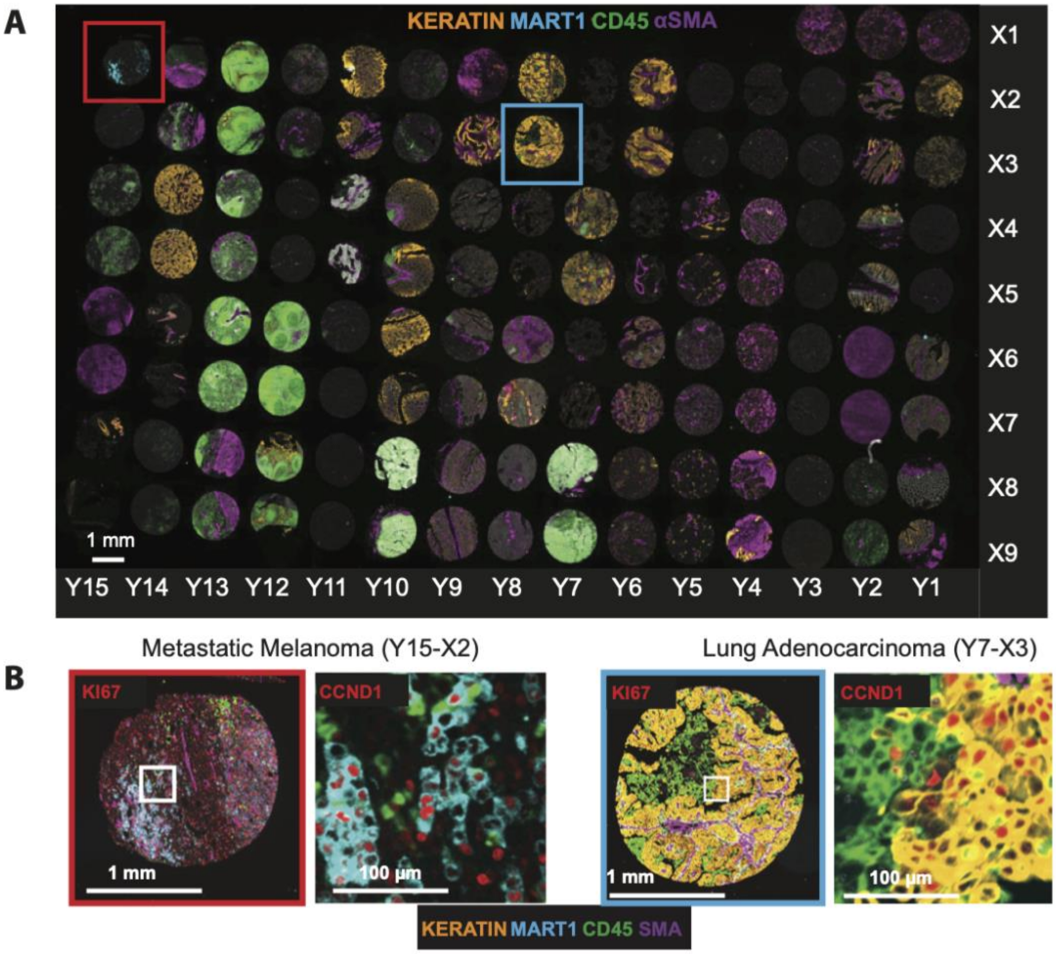

A. CyCIF whole slide image of EMIT visualizing Hoechst 33342-stained nuclear DNA (white), Keratin (orange), MART1 (cyan), CD45 (green) and SMA (purple). **B.** A zoom-in view of a metastatic melanoma (left, red box) and a lung adenocarcinoma (right, blue box) core. The highest zoom level is highlighted with white boxes in the corresponding low magnification images. This experiment was performed once.

Figure S2

Principal component analysis (PCA) of Spatial Feature Tables derived from EMIT images

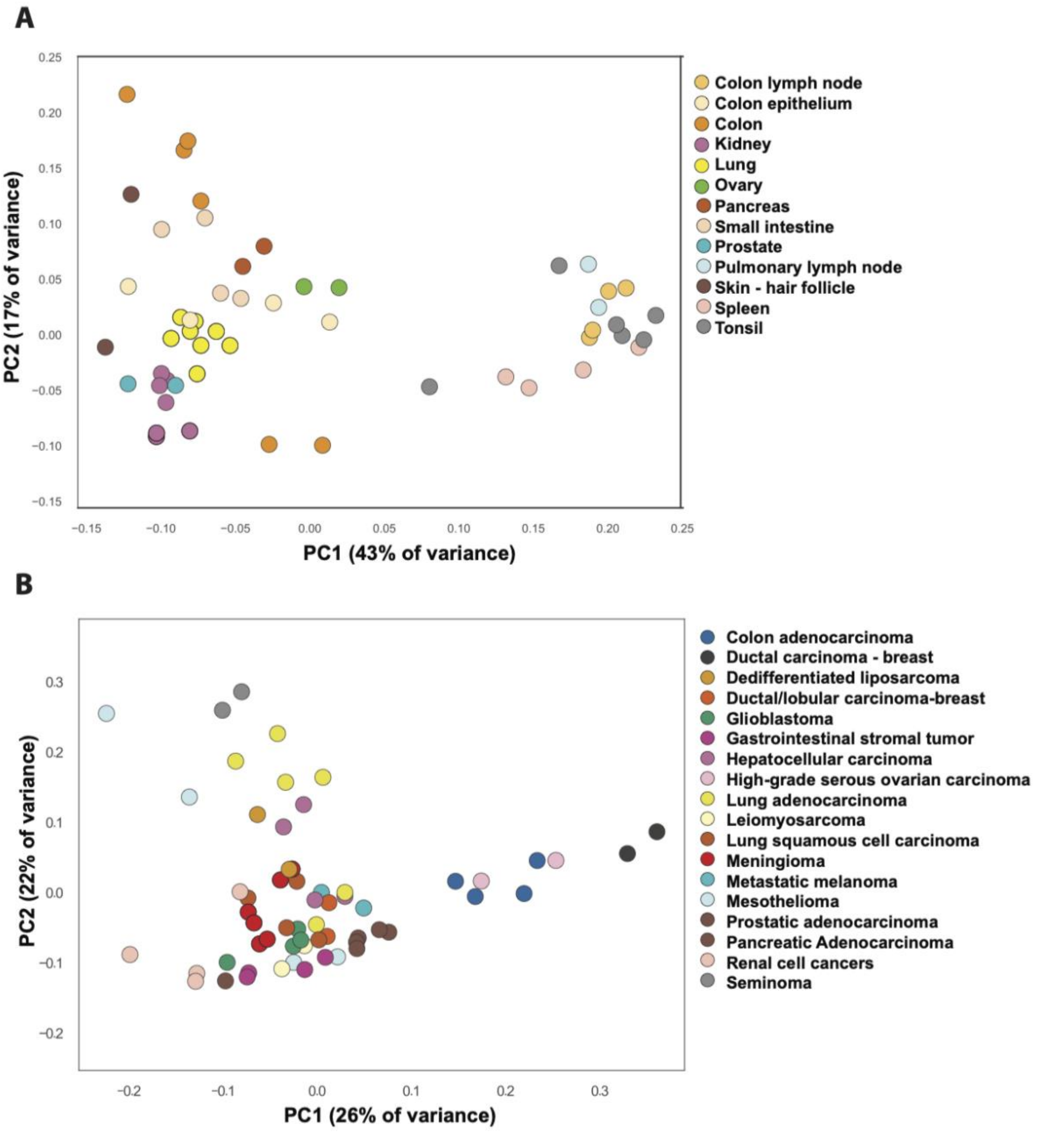

A. represents normal tissues and **B.** cancer tissues. Independent cores cluster to a substantial degree by tissue or cancer type; some variation is expected because tumors had different grades and derive from different individuals. Data from the following antibodies was used to generate the data: CD73, MART1, KI67, pan-cytokeratin, CD45, ECAD, α-SMA, CD32, CDKN1A, CCNA2, CDKN1C, CDKN1B, CCND1, cPARP, CCNB1, PCNA and CDK2.

Figure S3

Nextflow enables reproducible data processing using the provenance module

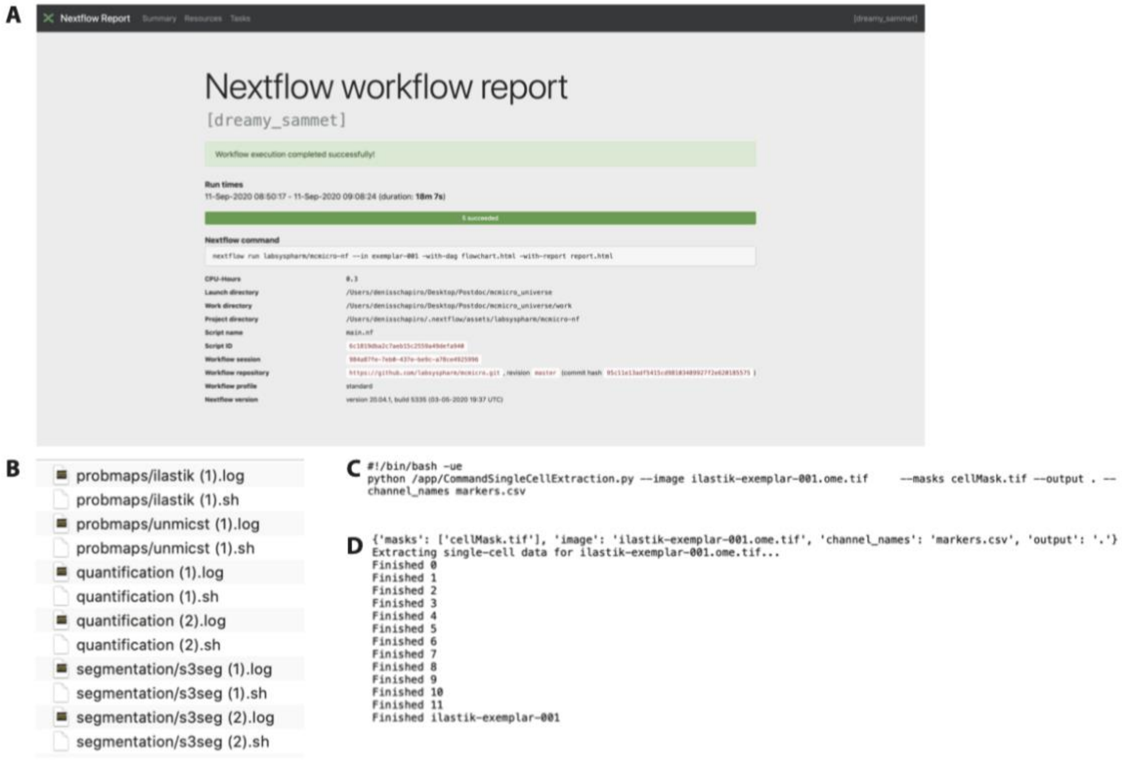

A. Nextflow report provides detailed documentation for used resources, directories, repositories (including commit hash) and the corresponding execution times. The report is browser based and interactive. **B-D.** Provenance reconstruction enabled by recording each executed command (.sh) and its output (.log). Representative examples of a command and its output are shown in **C** and **D**, respectively.

Figure S4

Detailed insight into the computational resources required by each module, generated by Nextflow

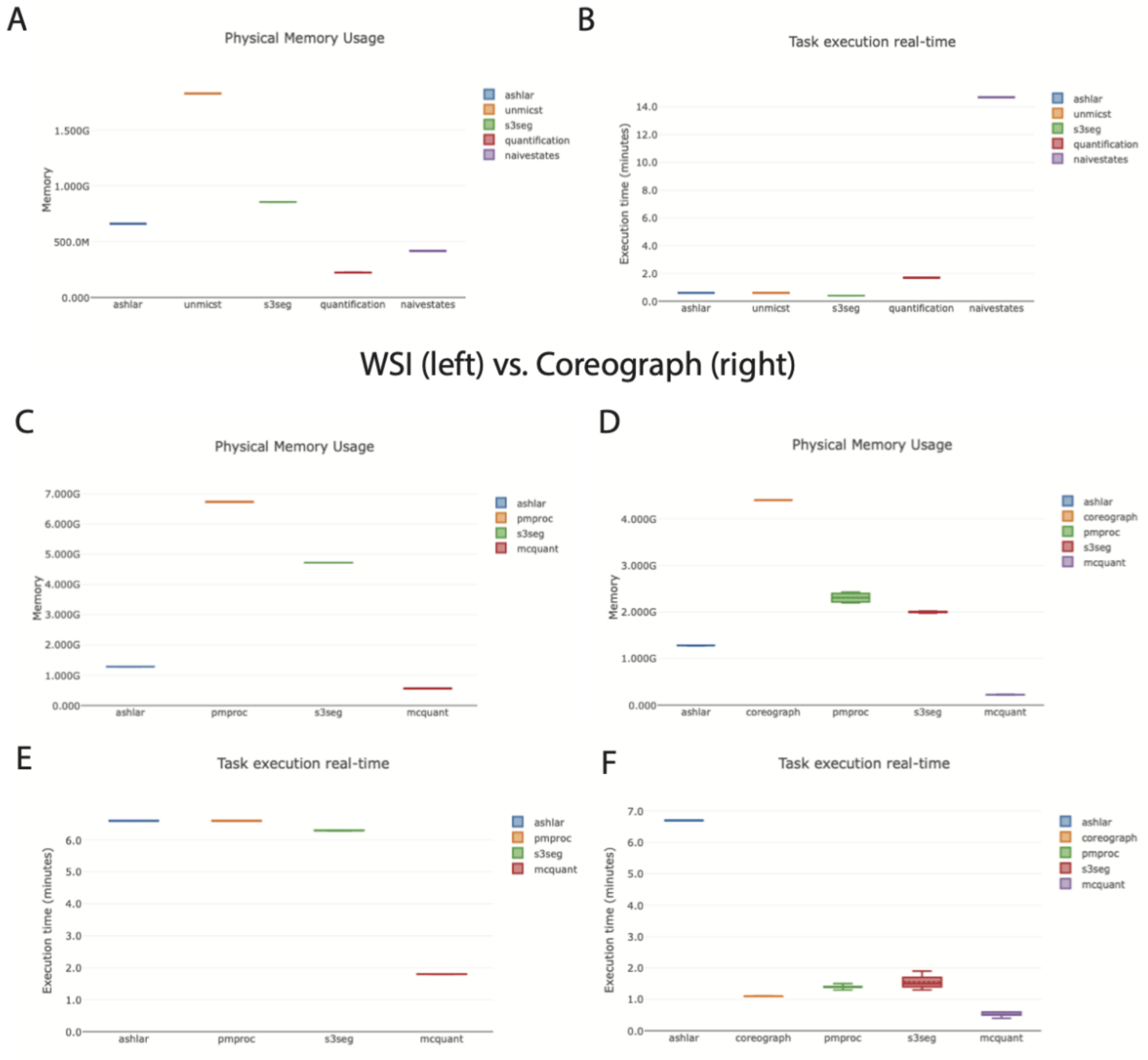

The data is viewed as an interactive browser-based report. **A.** Physical memory usage is recorded as either RAM only (visualized), RAM + Disk swap or % RAM allocated. **B.** Job duration is recorded as either execution time (visualized) or % time allocated. **C-D** compares the physical memory usage and **E-F** the task execution time in minutes between whole slide image TMA downstream processing (left, C and E) and Coreograph segmented TMAs (right, D and F). Panels **A, B, C** and **E** were derived when processing a single whole-slide image (n=1), while panels **D** and **F** correspond to processing the EMIT TMA (n=123 images). In all panels, the box shows inter-quartile range (q1 to q3) with a horizontal line denoting the median; the whiskers extend to the minimum and the maximum values.

Figure S5

The web-based Galaxy platform for Multiplexed Tissue Imaging analysis

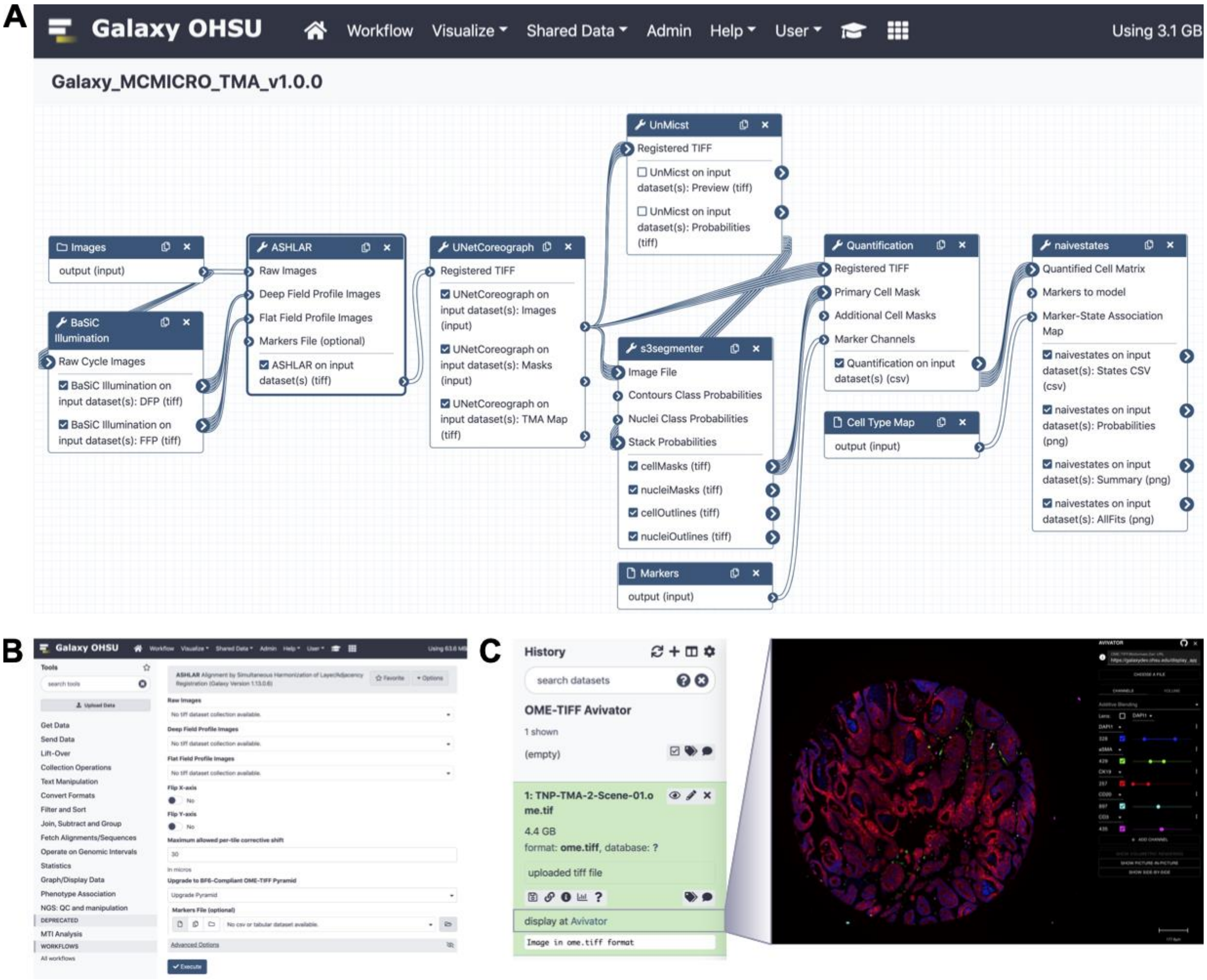

A. A detailed representation of the Galaxy interface implemented for MCMICRO. Process of a TMA is shown, including image registration, de-arraying, segmentation, quantification, and cell state phenotyping. A single line denotes one dataset flowing from the output of one tool to the input of the next tool, whereas multiple lines denote a group of datasets flowing from one tool to the next. **B**. Galaxy tool user interface showing the ASHLAR module. With this interface, a user can select tool inputs, set parameter values, and execute the tool all in a web browser. Default parameters are provided that can be changed at run time. **C.** Multi-channel images can be viewed interactively in a web browser from Galaxy using the Avivator visualization. A single core of the TMA with channels corresponding to Hoechst 33342-stained nuclear DNA (blue), α-smooth muscle actin (α-SMA; green), cytokeratin (red), B cell marker CD20 (cyan), and the T cell marker CD3 (magenta). With Avivator, users can browse and zoom around images, show and hide channels, and set channel colors and intensity ranges.

Supplementary Table 1: Highly multiplexed imaging methods

**Cyclic methods, fluorescence imaging**		**Cyclic immuno-histochemistry**	**Non-cyclic mass spectrometry based**
Co-detection by indexing **(CODEX)**[1]	Multiplexed Immunofluorescence **(MxIF)**[2]	Fluorescent Multiplex Immunohistochemistry **(mIHC)**[3]	Imaging Mass Cytometry (IMC)[4]
Immunostaining with signal amplification by exchange reaction (Immuno-SABER)[5]	Cyclic immunofluorescence **(CyCIF)**[6]	Multiplexed Immunohistochemical Consecutive Staining on Single Slide (MICSSS)[7]	Multiplexed Ion Beam Imaging (MIBI)[8]
	Iterative indirect immunofluorescence imaging (4i)[9]		

Table S1: Data collected in the course of this study using methods in bold were successfully processed by MCMICRO; results are shown in **Figure 2** and detailed descriptions of intermediate processing results are provided with the documentation available at mcmicro.org. Publicly available data for IMC and MIBI were also processed.

Supplementary Table 2: Available open-source tools for single cell analysis, image processing and visualization

Software	Scalable	Whole slide processing	Stitching and registration	Modular	Segmentation	Analysis	GUI
MCMICRO	Yes	Yes	Yes	Yes	Yes	Yes	No
Cytokit[10]	Yes	No (tiles)	No	No	Yes	Yes	Yes
starfish[11]	Yes	No (tiles)	No	Yes	Yes	Yes	No
histoCAT[12]	No	Yes	No	Yes	No	Yes	Yes
QuPath[13]	No	Yes	No	No	Yes	Yes	Yes
CytoMAP[14]	No	Yes	No	No	No	Yes	Yes
Facetto[15]	No	Yes	No	No	No	Yes	Yes
napari[16]	Visualization tool						
OMERO[17]	Visualization tool						
Minerva[18]	Visualization tool						
Seurat[19]	R toolkit for single cell genomics						
Scanpy[20]	Python toolkit for single cell genomics						
Cumulus[21]	Cloud-based framework for single cell genomics						

Table S2: List of open-source tools available for highly multiplexed image processing.

Supplementary Table 3: Detailed information about antibodies used to acquire the first EMIT image of a 123-core Tissue Microarray.

channel_number	cycle_number	marker_name	marker	Vendor	Cat. No.	Dilution	RRID	QC
1	1	DNA1	DNA					Pass
2	1	bg2b	Rabbit IgG	Thermo Fisher	A11070	2000	AB_2534114	NA
3	1	bg3b	Goat IgG	Thermo Fisher	A21432	2000	AB_2535853	NA
4	1	bg4b	Mouse IgG	Thermo Fisher	A21237	2000	AB_2535806	NA
5	2	DNA2	DNA					Pass
6	2	chREBP	chREBP	Abcam	ab92809	100	AB_10562135	Exploratory
7	2	CMA1	Mast Cell Chymase	Abcam	ab111239	100	AB_10863662	Pass
8	2	MBP	Major Basic Protein	BioRad	MCA5751	50	AB_10671914	Exploratory
9	3	DNA3	DNA					Pass
10	3	ECAD	E-Cadherin	CST	3199S	300	AB_10691457	Pass
11	3	panCK	pan-Cytokeratin	Thermo-Fisher	41-9003-82	1000	AB_11218704	Pass
12	3	CD45	CD45	BioLegend	304056	300	AB_2564155	Pass
13	4	DNA4	DNA					Pass
14	4	CD4	CD4	R&D Systems	FAB8165G	200	AB_2728839	Pass
15	4	CD3D	CD3d	abcam	ab208514	150	AB_2728789	Pass
16	4	CD8A	CD8a	invitrogen	50-0008-82	300	AB_2574149	Pass
17	5	DNA5	DNA					Pass
18	5	pJUN	p-cJun	CST	12714S	100	AB_2798004	Exploratory
19	5	cCASP3	Cleaved Caspase 3	CST	9978S	200	AB_10831820	Fail
20	5	IRF3_1	IRF3	SC	sc-33641 AF647	200	AB_2890146	Exploratory
21	6	DNA6	DNA					Pass
22	6	NUP133	NUP133	SC	sc-376763 AF488	100	AB_2889360	Pass
23	6	CALP	Calponin	Abcam	ab208051	200	AB_2890147	Exploratory
24	6	NUP98	NUP98	CST	13393S	200	AB_2728831	Pass
25	7	DNA7	DNA					Pass
26	7	LMNB1	LMNB1	abcam	ab194106	200	AB_2728786	Pass
27	7	IRF3_2	IRF3	CST	73147S	150	AB_2799834	Exploratory
28	7	CD45R	CD45R	BioLegend	103226	200	AB_389330	Fail
29	8	DNA8	DNA					Pass
30	8	IRF3_3	IRF3	abcam	ab204647	150	AB_2890152	Exploratory
31	8	PML	PML	abcam	ab217524	300	AB_2890149	Pass
32	8	GLUT1	GLUT1	abcam	ab195020	300	AB_2783877	Pass
33	9	DNA9	DNA					Pass
34	9	LMNAC	LaminAC	CST	8617S	100	AB_10997529	Pass
35	9	CD1B	CD1B	Biolegend	329108	100	AB_10613102	Fail
36	9	Hsp27	Hsp27	abcam	ab194078	1000	AB_2889283	Pass
37	10	DNA10	DNA					Pass
38	10	GAPDH	GAPDH	abcam	ab204276	500	AB_2889198	Pass
39	10	HSP90	HSP90	CST	70657S	300	AB_2799788	Exploratory
40	10	S100A11	S100A11	abcam	ab207545	300	AB_2889311	Pass
41	11	DNA11	DNA					Pass
42	11	TDP43	TDP43	abcam	ab193842	200	AB_2889241	Pass
43	11	OGT	OGT	abcam	ab215809	300	AB_2889304	Pass
44	11	COLL4	Collagen IV	eBioscience	51-9871-80	500	AB_10854267	Pass
45	12	DNA12	DNA					Pass
46	12	FCERG1A	FCERG1A	SC	sc-517280 AF488	100	AB_2889358	Fail
47	12	SQSTM1	SQSTM1	Abcam	ab203430	100	AB_2728795	Fail
48	12	LMNB2	LMNB2	Abcam	ab200427	200	AB_2889288	Pass
49	13	DNA13	DNA					Pass
50	13	HSP70	Hsp70	abcam	ab61907	200	AB_2120890	Fail
51	13	KI67	KI67	Thermo-Fisher	41-5698-82	300	AB_11220285	Fail
52	13	pH2AX	pH2AX	BioLegend	613407	200	AB_2114994	Pass
53	14	DNA14	DNA					Pass
54	14	SEC61A	SEC61A	abcam	ab205794	100	AB_2890150	Pass
55	14	EPCAM	EpCAM	BioLegend	324205	200	AB_756079	Pass
56	14	CD86	CD86	eBioscience	62-0869-41	100	AB_2637394	Fail
57	15	DNA15	DNA					Pass
58	15	LMNAC_2	Lamin A/C	Abcam	ab185014	100	AB_2889225	Pass
59	15	ECAD	E-Cadherin	abcam	ab206878	300	AB_2801591	Pass
60	15	KI67	Ki67	BioLegend	350509	200	AB_10900821	Fail
61	16	DNA16	DNA					Pass
62	16	LBR	Lamin B Receptor	Abcam	ab201532	100	AB_2889290	Pass
63	16	VEGFR2	VEGFR2	CST	12634S	150	AB_2728829	Pass
64	16	TUBG	Gamma tubulin	Abcam	ab191114	100	AB_2889219	Pass

Supplementary Table 4: Detailed information about antibodies used to acquire the second EMIT image of a 123-core Tissue Microarray.

channel_number	cycle_number	marker_name	marker	Vendor	Catalog Number	Dilution	RRID	QC
1	1	DNA1	DNA	NA	NA	NA	NA	Pass
2	1	Rabbit IgG	Rabbit IgG	Thermo Fisher	A11070	2000	AB_2534114	NA
3	1	Goat IgG	Goat IgG	Thermo Fisher	A21432	2000	AB_2535853	NA
4	1	Mouse IgG	Mouse IgG	Thermo Fisher	A21237	2000	AB_2535806	NA
5	2	DNA2	DNA	NA	NA	NA	NA	Pass
6	2	CD73	CD73	CST	13160S	200	AB_2716625	Pass
7	2	CD107B	CD107B	R&D Systems	AF6228	500	AB_10971818	Exploratory
8	2	MART1	MART1	BioLegend	917901	300	AB_2565202	Pass
9	3	DNA3	DNA	NA	NA	NA	NA	Pass
10	3	KI67	KI67	CST	11882S	200	AB_2687824	Pass
11	3	pan-CK	pan-CK	Thermo-Fisher	41-9003-82	1000	AB_11218704	Pass
12	3	CD45	CD45	BioLegend	304056	300	AB_2564155	Pass
13	4	DNA4	DNA	NA	NA	NA	NA	Pass
14	4	ECAD	ECAD	Cell Signaling Technology	3199S	300	AB_10691457	Pass
15	4	aSMA	aSMA	abcam	ab202509	1000	AB_2868435	Pass
16	4	CD56	CD56	SC	sc-7326 AF647	100	AB_2890151	Fail
17	5	DNA5	DNA	NA	NA	NA	NA	Pass
18	5	CD13	CD13	Abcam	ab239271	150	AB_2889257	Fail
19	5	CD63	CD63	Abcam	ab215431	150	AB_2889263	Exploratory
20	5	CD32	CD32	Abcam	ab209451	150	AB_2889260	Exploratory
21	6	DNA6	DNA	NA	NA	NA	NA	Pass
22	6	CDKN1A	CDKN1A	CST	5487S	200	AB_10699024	Pass
23	6	CCNA2	CCNA2	Abcam	ab217731	300	AB_2884890	Pass
24	6	CDKN1C	CDKN1C	Abcam	ab199253	200	AB_2889305	Pass
25	7	DNA7	DNA	NA	NA	NA	NA	Pass
26	7	PCNA_1	PCNA	BioLegend	307909	500	AB_2160546	Pass
27	7	pAUR	pAUR	CST	13464S	300	AB_2728824	Fail
28	7	CDKN1B_1	CDKN1B	Abcam	ab194234	300	AB_2728802	Pass
29	8	DNA8	DNA	NA	NA	NA	NA	Pass
30	8	CCND1	CCND1	Abcam	AB190194	200	AB_2728784	Pass
31	8	cPARP	cPARP	CST	6894S	100	AB_10830735	Pass
32	8	CDKN1B_2	CDKN1B	Abcam	ab194234	300	AB_2728802	Pass
33	9	DNA9	DNA	NA	NA	NA	NA	Pass
34	9	pCREB	pCREB	CST	9187S	150	AB_659957	Pass
35	9	CCNB1	CCNB1	Abcam	ab214381	300	AB_2884889	Pass
36	9	CCNE	CCNE	TF	50-9714-80	200	AB_2574350	Fail
37	10	DNA10	DNA	NA	NA	NA	NA	Pass
38	10	PCNA_2	PCNA	CST	8580S	1000	AB_11178664	Pass
39	10	CDK2	CDK2	Abcam	ab208043	300	AB_2889266	Pass
40	10	CDKN2A	CDKN2A	Abcam	ab192054	500	AB_2889194	Fail

Supplementary table references:

1. Goltsev, Y., Samusik, N., Kennedy-Darling, J., Bhate, S., Hale, M., Vazquez, G., Black, S. & Nolan, G. P. Deep Profiling of Mouse Splenic Architecture with CODEX Multiplexed Imaging. *Cell* **174,** 968-981.e15 (2018).
2. Gerdes, M. J., Sevinsky, C. J., Sood, A., Adak, S., Bello, M. O., Bordwell, A., Can, A., Corwin, A., Dinn, S., Filkins, R. J., Hollman, D., Kamath, V., Kaanumalle, S., Kenny, K., Larsen, M., Lazare, M., Li, Q., Lowes, C., McCulloch, C. C., McDonough, E., Montalto, M. C., Pang, Z., Rittscher, J., Santamaria-Pang, A., Sarachan, B. D., Seel, M. L., Seppo, A., Shaikh, K., Sui, Y., Zhang, J. & Ginty, F. Highly multiplexed single-cell analysis of formalin-fixed, paraffin-embedded cancer tissue. *PNAS* **110,** 11982–11987 (2013).
3. Tsujikawa, T., Kumar, S., Borkar, R. N., Azimi, V., Thibault, G., Chang, Y. H., Balter, A., Kawashima, R., Choe, G., Sauer, D., El Rassi, E., Clayburgh, D. R., Kulesz-Martin, M. F., Lutz, E. R., Zheng, L., Jaffee, E. M., Leyshock, P., Margolin, A. A., Mori, M., Gray, J. W., Flint, P. W. & Coussens, L. M. Quantitative Multiplex Immunohistochemistry Reveals Myeloid-Inflamed Tumor-Immune Complexity Associated with Poor Prognosis. *Cell Reports* **19,** 203–217 (2017).
4. Giesen, C., Wang, H. A. O., Schapiro, D., Zivanovic, N., Jacobs, A., Hattendorf, B., Schüffler, P. J., Grolimund, D., Buhmann, J. M., Brandt, S., Varga, Z., Wild, P. J., Günther, D. & Bodenmiller, B. Highly multiplexed imaging of tumor tissues with subcellular resolution by mass cytometry. *Nat Methods* **11,** 417–422 (2014).
5. Saka, S. K., Wang, Y., Kishi, J. Y., Zhu, A., Zeng, Y., Xie, W., Kirli, K., Yapp, C., Cicconet, M., Beliveau, B. J., Lapan, S. W., Yin, S., Lin, M., Boyden, E. S., Kaeser, P. S., Pihan, G., Church, G. M. & Yin, P. Immuno-SABER enables highly multiplexed and amplified protein imaging in tissues. *Nat Biotechnol* **37,** 1080–1090 (2019).
6. Lin, J.-R., Izar, B., Wang, S., Yapp, C., Mei, S., Shah, P. M., Santagata, S. & Sorger, P. K. Highly multiplexed immunofluorescence imaging of human tissues and tumors using t-CyCIF and conventional optical microscopes. *eLife* **7,** e31657 (2018).
7. Remark, R., Merghoub, T., Grabe, N., Litjens, G., Damotte, D., Wolchok, J. D., Merad, M. & Gnjatic, S. In-depth tissue profiling using multiplexed immunohistochemical consecutive staining on single slide. *Science Immunology* **1,** aaf6925–aaf6925 (2016).
8. Angelo, M., Bendall, S. C., Finck, R., Hale, M. B., Hitzman, C., Borowsky, A. D., Levenson, R. M., Lowe, J. B., Liu, S. D., Zhao, S., Natkunam, Y. & Nolan, G. P. Multiplexed ion beam imaging (MIBI) of human breast tumors. *Nat Med* **20,** 436–442 (2014).
9. Gut, G., Herrmann, M. D. & Pelkmans, L. Multiplexed protein maps link subcellular organization to cellular states. *Science* **361,** eaar7042 (2018).

10. Czech, E., Aksoy, B. A., Aksoy, P. & Hammerbacher, J. Cytokit: a single-cell analysis toolkit for high dimensional fluorescent microscopy imaging. *BMC Bioinformatics* **20,** 448 (2019).

11. *https://spacetx-starfish.readthedocs.io/en/latest/*.

12. Schapiro, D., Jackson, H. W., Raghuraman, S., Fischer, J. R., Zanotelli, V. R. T., Schulz, D., Giesen, C., Catena, R., Varga, Z. & Bodenmiller, B. histoCAT: analysis of cell phenotypes and interactions in multiplex image cytometry data. *Nat Methods* **14,** 873–876 (2017).

13. Bankhead, P., Loughrey, M. B., Fernández, J. A., Dombrowski, Y., McArt, D. G., Dunne, P. D., McQuaid, S., Gray, R. T., Murray, L. J., Coleman, H. G., James, J. A., Salto-Tellez, M. & Hamilton, P. W. QuPath: Open source software for digital pathology image analysis. *Sci Rep* **7,** 16878 (2017).

14. Stoltzfus, C. R., Filipek, J., Gern, B. H., Olin, B. E., Leal, J. M., Wu, Y., Lyons-Cohen, M. R., Huang, J. Y., Paz-Stoltzfus, C. L., Plumlee, C. R., Pöschinger, T., Urdahl, K. B., Perro, M. & Gerner, M. Y. CytoMAP: A Spatial Analysis Toolbox Reveals Features of Myeloid Cell Organization in Lymphoid Tissues. *Cell Reports* **31,** 107523 (2020).

15. Krueger, R., Beyer, J., Jang, W.-D., Kim, N. W., Sokolov, A., Sorger, P. K. & Pfister, H. Facetto: Combining Unsupervised and Supervised Learning for Hierarchical Phenotype Analysis in Multi-Channel Image Data. *IEEE Trans. Visual. Comput. Graphics* **26,** 227–237 (2020).

16. Sofroniew, N., Talley Lambert, Evans, K., Nunez-Iglesias, J., Yamauchi, K., Solak, A. C., Buckley, G., Bokota, G., Tung, T., Ziyangczi, Freeman, J., Boone, P., Winston, P., Loic Royer, Har-Gil, H., Axelrod, S., Rokem, A., Bryant, Hector, Mars Huang, Pranathi Vemuri, Dunham, R., Jakirkham, Siqueira, A. D., Bhavya Chopra, Wood, C., Gohlke, C., Bennett, D., DragaDoncila & Perlman, E. *napari/napari: 0.3.5*. (Zenodo, 2020). doi:10.5281/ZENODO.3555620

17. Allan, C., Burel, J.-M., Moore, J., Blackburn, C., Linkert, M., Loynton, S., Macdonald, D., Moore, W. J., Neves, C., Patterson, A., Porter, M., Tarkowska, A., Loranger, B., Avondo, J., Lagerstedt, I., Lianas, L., Leo, S., Hands, K., Hay, R. T., Patwardhan, A., Best, C., Kleywegt, G. J., Zanetti, G. & Swedlow, J. R. OMERO: flexible, model-driven data management for experimental biology. *Nat Methods* **9,** 245–253 (2012).

18. Rashid, R., Chen, Y.-A., Hoffer, J., Muhlich, J. L., Lin, J.-R., Krueger, R., Pfister, H., Mitchell, R., Santagata, S. & Sorger, P. K. *Online narrative guides for illuminating tissue atlas data and digital pathology images*. (Scientific Communication and Education, 2020). doi:10.1101/2020.03.27.001834

19. Satija, R., Farrell, J. A., Gennert, D., Schier, A. F. & Regev, A. Spatial reconstruction of single-cell gene expression data. *Nat Biotechnol* **33,** 495–502 (2015).

20. Wolf, F. A., Angerer, P. & Theis, F. J. SCANPY: large-scale single-cell gene expression data analysis. *Genome Biology* **19,** (2018).

21. Li, B., Gould, J., Yang, Y., Sarkizova, S., Tabaka, M., Ashenberg, O., Rosen, Y., Slyper, M., Kowalczyk, M. S., Villani, A.-C., Tickle, T., Hacohen, N., Rozenblatt-Rosen, O. & Regev, A. Cumulus provides cloud-based data analysis for large-scale single-cell and single-nucleus RNA-seq. *Nat Methods* **17,** 793–798 (2020).
