# Supplementary material for: MCMICRO: a scalable, modular image-processing pipeline for multiplexed tissue imaging
Source: Nat Methods. 2021 Nov 25;19(3):311–5. doi: 10.1038/s41592-021-01308-y (PMC8916956; doi:10.1038/s41592-021-01308-y)
Supplement: Supplementary file 2 — Reporting Summary [file 41592_2021_1308_MOESM2_ESM.pdf]

nature portfolio

Corresponding author(s): Peter K. Sorger

Last updated by author(s): Aug 20, 2021

Reporting Summary

Nature Portfolio wishes to improve the reproducibility of the work that we publish. This form provides structure for consistency and transparency in reporting. For further information on Nature Portfolio policies, see our Editorial Policies and the Editorial Policy Checklist.

Statistics

For all statistical analyses, confirm that the following items are present in the figure legend, table legend, main text, or Methods section.

n/a	Confirmed	
☐	☒	The exact sample size (n) for each experimental group/condition, given as a discrete number and unit of measurement
☒	☐	A statement on whether measurements were taken from distinct samples or whether the same sample was measured repeatedly
☒	☐	The statistical test(s) used AND whether they are one- or two-sided<br>*Only common tests should be described solely by name; describe more complex techniques in the Methods section.*
☒	☐	A description of all covariates tested
☒	☐	A description of any assumptions or corrections, such as tests of normality and adjustment for multiple comparisons
☐	☒	A full description of the statistical parameters including central tendency (e.g. means) or other basic estimates (e.g. regression coefficient) AND variation (e.g. standard deviation) or associated estimates of uncertainty (e.g. confidence intervals)
☒	☐	For null hypothesis testing, the test statistic (e.g. F, t, r) with confidence intervals, effect sizes, degrees of freedom and P value noted<br>*Give P values as exact values whenever suitable.*
☒	☐	For Bayesian analysis, information on the choice of priors and Markov chain Monte Carlo settings
☒	☐	For hierarchical and complex designs, identification of the appropriate level for tests and full reporting of outcomes
☒	☐	Estimates of effect sizes (e.g. Cohen's d, Pearson's r), indicating how they were calculated

Our web collection on statistics for biologists contains articles on many of the points above.

Software and code

Policy information about availability of computer code

Data collection	CODEX Instrument Manager v1.29, CyteFinder v3.9.0.1
Data analysis	ImageJ (FIJI OpenJDK v8), ilastik v1.3.3, FastPG v3.10, ASHLAR v1.14.0, Coreograph v2.2.2, UnMicst v2.6.14, S3segmenter v1.3.5, SCIMAP v0.17.6, https://github.com/labsyspharm/mcmicro, https://github.com/labsyspharm/mcmicro_manuscript

For manuscripts utilizing custom algorithms or software that are central to the research but not yet described in published literature, software must be made available to editors and reviewers. We strongly encourage code deposition in a community repository (e.g. GitHub). See the Nature Portfolio guidelines for submitting code & software for further information.

Data

Policy information about availability of data

All manuscripts must include a data availability statement. This statement should provide the following information, where applicable:

- Accession codes, unique identifiers, or web links for publicly available datasets
- A description of any restrictions on data availability
- For clinical datasets or third party data, please ensure that the statement adheres to our policy

All data is publicly available at https://mcmicro.org/datasets.html and https://labsyspharm.github.io/HTA-CRCATLAS-1/index.html

Field-specific reporting

Please select the one below that is the best fit for your research. If you are not sure, read the appropriate sections before making your selection.

☒ Life sciences ☐ Behavioural & social sciences ☐ Ecological, evolutionary & environmental sciences

For a reference copy of the document with all sections, see nature.com/documents/nr-reporting-summary-flat.pdf

Life sciences study design

All studies must disclose on these points even when the disclosure is negative.

Sample size	Three consecutive sections of tonsil and colorectal cancer specimens were analyzed with 5 different technologies. One Tissue Microarray (TMA) with 123 cores was analyzed with CyCIF (EMIT dataset). The sample size was deemed sufficient, because processing every individual image gave rise to measurements for 10^5 to 10^6 single cells, which allowed for an effective comparison across tissues and imaging technologies.
Data exclusions	No data were excluded.
Replication	In whole-slide imaging, replication was approximated via consecutive sectioning. In the TMA, each tissue type was considered in at least 2 cores.
Randomization	The assignment of consecutive sections to imaging technologies was random. The assignment of tissues types to physical location on the TMA was also random.
Blinding	Blinding was not relevant, because the study focused on direct comparison across tissues and imaging platforms.

Reporting for specific materials, systems and methods

We require information from authors about some types of materials, experimental systems and methods used in many studies. Here, indicate whether each material, system or method listed is relevant to your study. If you are not sure if a list item applies to your research, read the appropriate section before selecting a response.

Materials & experimental systems

n/a	Involved in the study	
☐	☒	Antibodies
☒	☐	Eukaryotic cell lines
☒	☐	Palaeontology and archaeology
☒	☐	Animals and other organisms
☐	☒	Human research participants
☒	☐	Clinical data
☒	☐	Dual use research of concern

Methods

n/a	Involved in the study	
☒	☐	ChIP-seq
☒	☐	Flow cytometry
☒	☐	MRI-based neuroimaging

Antibodies

Antibodies used	Antibody information (including supplier name, catalog number and dilution) is included in Supplementary Tables 3 and 4.
Validation	All antibodies used are commercially available and have been validated by the corresponding vendors, with information provided on their websites. Additionally, all antibodies were re-validated in-house using the protocols described in previous publications [DOI: 10.7554/eLife.31657 and DOI: 10.1038/s41596-019-0206-y], with information and images provided at https://www.cycif.org/antibodies

Human research participants

Policy information about studies involving human research participants

Population characteristics	A de-identified tonsil specimen from a 4-year old Caucasian female was used for whole-slide imaging.
Recruitment	The tonsil specimen was procured from the Cooperative Human Tissue Network (CHTN), Western Division, as part of the Human Tumor Atlas (HTAN) SARDANA trans-network project (TNP).
Ethics oversight	Regulatory documents including Institutional Review Board (IRB - Brigham and Women's Hospital (BWH IRB 2018P001627)) protocols, data use agreements and tissue use agreements were in place to ensure regulatory compliance.

Note that full information on the approval of the study protocol must also be provided in the manuscript.
